# Floating wind turbines structural details fatigue life assessment

**DOI:** 10.1038/s41598-023-43554-4

**Published:** 2023-09-28

**Authors:** Oleg Gaidai, Vladimir Yakimov, Fang Wang, Fuxi Zhang, Rajiv Balakrishna

**Affiliations:** 1 Shanghai Ocean University, Shanghai, China; 2 Central Marine Research and Design Institute, Saint Petersburg, Russia; 3 University of Stavanger, Stavanger, Norway

**Keywords:** Energy infrastructure, Mechanical engineering

## Abstract

Fatigue damage prediction is essential for safety of contemporary offshore energy industrial projects, like offshore wind turbines, that are to be designed for sufficiently long operational period of time, with minimal operational disruptions. Offshore structures being designed to withstand environmental loadings due to winds and waves. Due to accumulated fatigue damage, offshore wind floating turbines may develop material cracks in their critical locations sooner than expected. Dataset needed for an accurate assessment of fatigue damage may be produced by either extensive numerical modeling, or direct measurements. However, in reality, temporal length of the underlying dataset being typically too short to provide an accurate calculation of direct fatigue damage and fatigue life. Hence, the objective of this work is to contribute to the development of novel fatigue assessment methods, making better use of limited underlying dataset. In this study, in-situ environmental conditions were incorporated to assess offshore FWT tower base stresses; then structural cumulative fatigue damage has been assessed. Novel deconvolution extrapolation method has been introduced in this study, and it was shown to be able to accurately predict long-term fatigue damage. The latter technique was validated, using artificially reduced dataset, and resulted in fatigue damage that was shown to be close to the damage, calculated from the full original underlying dataset. Recommended method has been shown to utilize available dataset much more efficiently, compared to direct fatigue estimation. Accurate fatigue assessment of offshore wind turbine structural characteristics is essential for structural reliability, design, and operational safety.

## Introduction

With more wind turbines being designed and installed, wind energy is taking the lead in a field of renewable energy power generation. Wind energy is cost-free and accessible, having quite attractive energy potential. Installation of new, larger wind turbines has relocated offshore, due to accessible space, offers greater offshore wind potential, helping to achieve net-zero emission targets by 2050^1^. No greenhouse gases, such as CO_2_, nitrogen oxide, sulphur oxide, being emitted during FWT (floating wind turbine) operation. FWT market expands quite dynamically, especially for developing countries, with increasing energy demands, onshore wind, and even more for offshore wind, boosting national energy sustainability. According to the IRENA (International Renewable Energy Agency), offshore wind capacity factors will increase between 40 and 60% worldwide^1^. Total installed capacity of wind energy exceeded 700 GW in 2022, robust increase of 15% over 2019. The Hywind Scotland project, being first floating wind farm in the world, has already exceeded 50% capacity factor in its first 2 years of successful operation. As more FWTs developed, and put into service, there is rising necessity to design new FWTs more economically, while maintaining safety margins, and operational readiness levels. To improve design, minimize failures, reduce FWT downtime due to maintenance, accurate load prediction methods being required for aerodynamic, control, as well as dynamical modelling of FWT.

**Figure 1:**
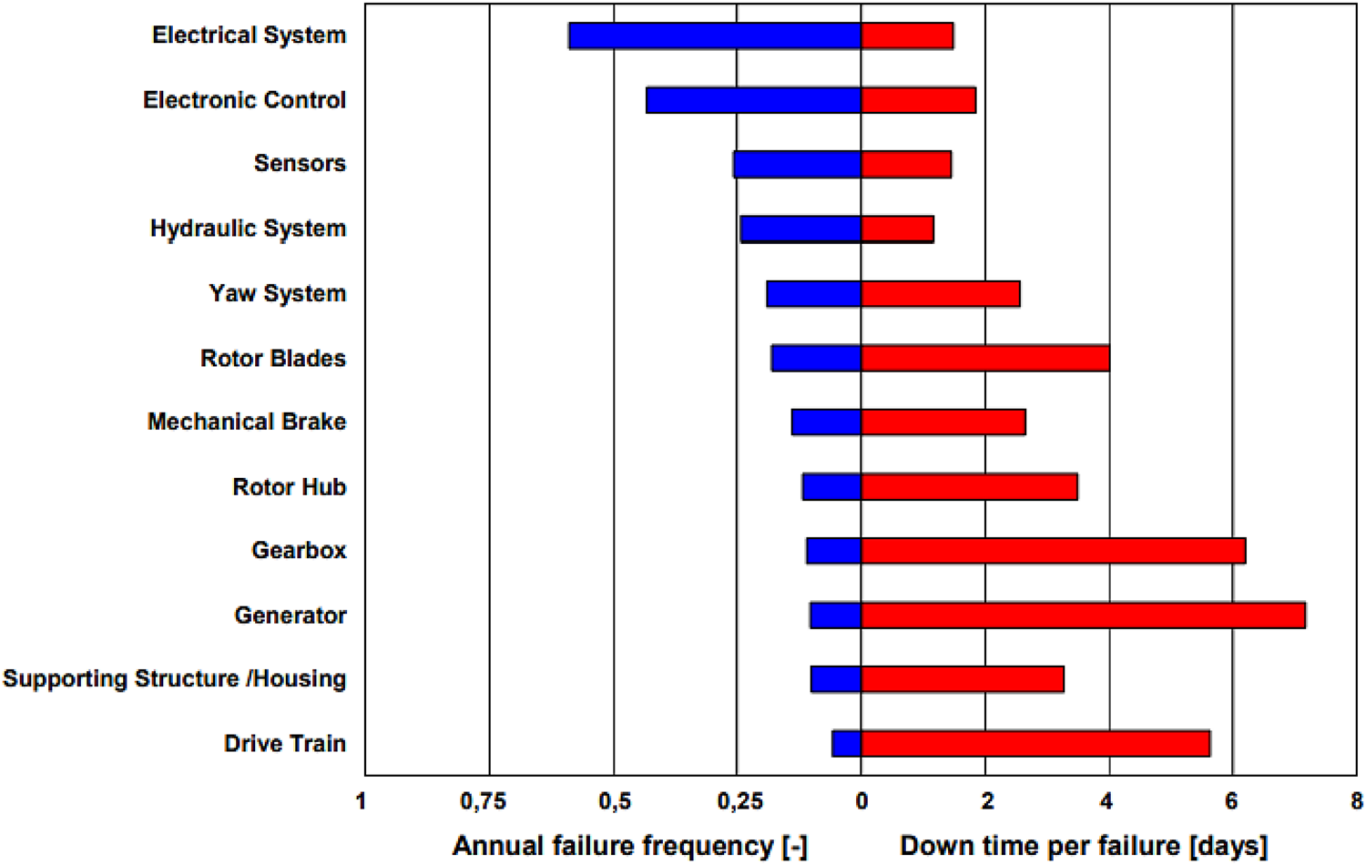
Reliability and downtime for FWT sub-assemblies^1^.

Bottom fixed mono-pile wind farms nowadays making up majority of offshore wind farms; however, bottom-fixed wind turbines cannot be yet made to be profitable^1–3^. In deep seas, where 80% of FWTs being installed, FWTs offer substantial advantages. In the near future 10-MW FWTs will be prioritized, hence, this study aims to contribute to reliability aspects of such FWT critical parts. In the past 2 decades, FWT's size was steadily increasing to save costs; as FWTs capacity increased, new technological developments have been required to develop crucial FWT parts including the motor, blades, controls. Majority of FWTs, however, didn't survive as long, as they were expected to, having negative effect on FWT operational expenses. According to studies on FWT drivetrain status monitoring, the gearbox in the drivetrain caused one of the highest downtimes per-failure^4^, see Fig. 1.

**Figure 2:**
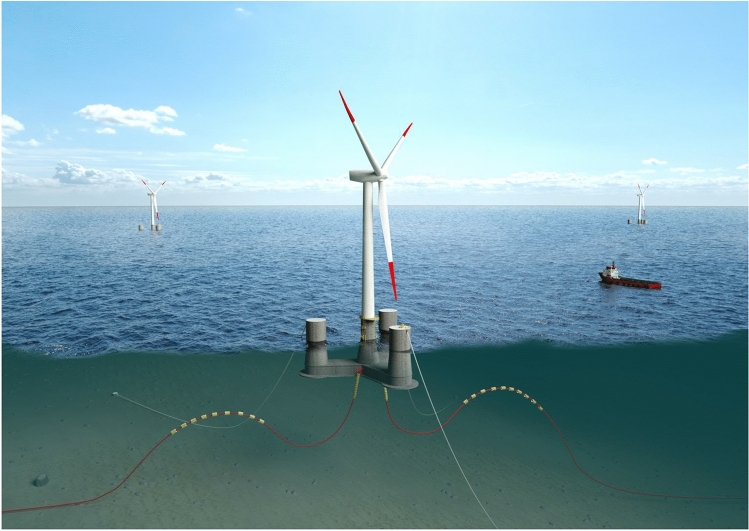
OO-Star FWT semi-submersible 10 MW concept^2^.

To develop efficient numerical methods for estimating the effects of waves and winds on FWTs, numerous researches were carried out. FWT structural details extreme dynamics as well as fatigue damage known to exhibit complex nonlinear cross-correlated nature^4^. FWT drivetrain dynamics of a 750KW spar type FWT has been analysed in^5–8^, for more details on FWT drivetrain design, see^9–12^. FWT drivetrains typically being subjected to more volatile load uncertainties, compared to those of land-based turbines, due to more complex offshore-environmental wind-wave loading nature. Figure 2 presents OO-Star FWT semi-submersible 10-MW design concept.

To identify structural load reactions, a detailed understanding of the FWT drivetrains' dynamic behaviour is required, in order to decrease fault rate, increase service (fatigue) life, yet save operational costs. Within FWT hydrodynamic study, irregular waves and water depth being 2 separate concepts, since FWTs are frequently placed in shallow water areas, where waves become more nonlinear, creating significant increase in hydrodynamic loads. 2 methods may be used to evaluate extreme FWT loads: 1st strategy is to simulate for example 10,000-year extreme environmental condition, leading to a single excessive structural load dataset; 2nd strategy, is to perform long-term statistical analysis, based on various environmental condition, according to in-situ scatter diagram. This study advocates 2nd approach, as being more accurate and representative of in-situ environment.

Development of novel FWT structural reliability tools will serve future better design of FWT control mechanisms, preventing FWT mechanical damage. Application of multivariate extreme value theory (EVT) being important from both practical engineering and design standpoints, since there are non-negligible nonlinear correlations between different FWT system components. Methodology, advocated in this study offers practical way of using relatively small nonstationary dataset effectively, and then subsequently assess FWT structural failure or damage risks. This study promotes MC (Monte Carlo) based statistical methodology, that may naturally incorporate underlying FWT structural nonlinear effects. For comparisons between suggested method and those that have been previously benchmarked in various applications, see^8–17^. Among key advantages of the advocated methodology, is that it does not directly rely on asymptotic EVT assumptions, therefore it introduces fewer residual inaccuracies in the final failure risks predictions.

While continuing to optimize parametric models and probabilistic techniques, writers conducted a detailed uncertainty analysis^7^. In^8^ authors sought to simplify and consolidate the many strategies mentioned above. Numerous studies on more precise estimations of the structural fatigue damage of FWTs have recently been conducted^12^, where authors used statistical and modeling techniques to quantify the long-term fatigue damage/life of offshore FWTs. Additional studies are needed to develop novel methods that are able to work with limited, non-stationary datasets, with classic methods to be used for cross-validation of new proposed reliability methods. The authors of this study have previously proposed four parameter Weibull method, extrapolating probability distribution function (PDF) tails (region with high response or load levels)^18^. The latter technique was used to extrapolate PDF tails using modified (four parameter) Weibull PDF and the mean up-crossing rate function, but not the tail of fatigue-related VM (von Mises) stress range distribution.

Distinctive advantage of this study, compared to existing fatigue life assessment methods, is that advocated methodology makes it possible to assess fatigue life fairly accurately, using even limited underlying dataset, taking into account stress cycles PDF tail.

## Dynamic system

**Figure 3:**
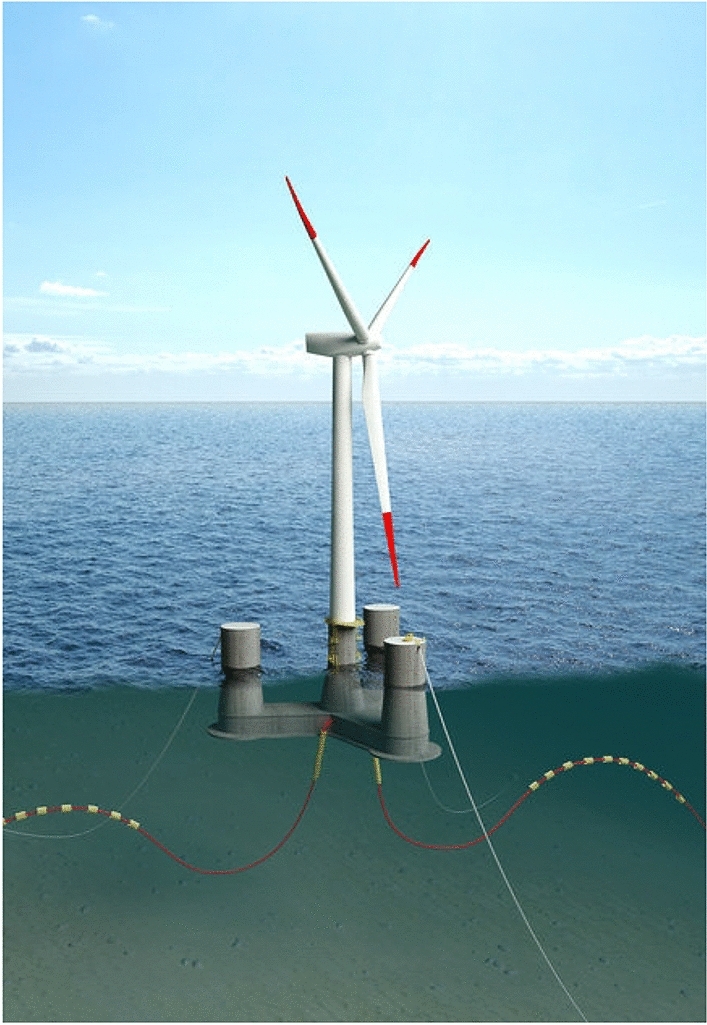
10-MW OO-Star FWT^13^.

A 10-MW floating wind turbine (FWT) dynamic system^13^ has been used in this study, as illustrated in Fig. 3.

In the next Section the reference FWT will be briefly described.

### DTU 10-MW reference FWT

A 10-MW reference wind turbine (RWT) constructed from an NREL 5-MW RWT has been used in this investigation. The RWT is a typical three-bladed, clockwise rotating, upwind FWT controlled by collective pitch and variable speed. This FWT was created in compliance with the Class 1A wind regime specified by IEC (the International Electrotechnical Commission). The DTU (Danish Technical University) 10-MW RWT numerical model has been successfully developed and studied in a number of scholarly articles^19–22^. The brief DTU 10-MW RWT summary is presented in Table 1.

**Table 1:** DTU 10-MW reference FWT key parameters^13^.

Parameters	Values
Rating	10-MW
Type	Upwind/3 blades
Control	Collective pitch, variable-speed
Drivetrain	Multiple stage gearbox, medium-speed
Cut-in, rated and cut-out wind speed (m/s)	4, 11.4, 25
Minimum and maximum rotor speed (rpm)	6.0, 9.6
Max generator speed (rpm)	480
Rotor diameter (m)	178.3
Hub height (m)	119.0
Rotor mass (tones)	228.2
Nacelle mass (tones)	446.0
Tower mass (tones)	1257.1

### OO-Star semi-submersible FWT

Semi-submersible floating structure typically acts as support for 10-MW RWT. It was first introduced by Olav Olsen AS in the LIFES 50 + project^13^. Three external columns surround a center column in the post-tensioned concrete floater; 4 columns are fastened to the bottom of the slab-attached pontoon, which has a star-shaped form. Three catenary mooring lines that each have a clumped mass connected that separates them into 2 portions keep the FWT floater in place.

**Figure 4:**
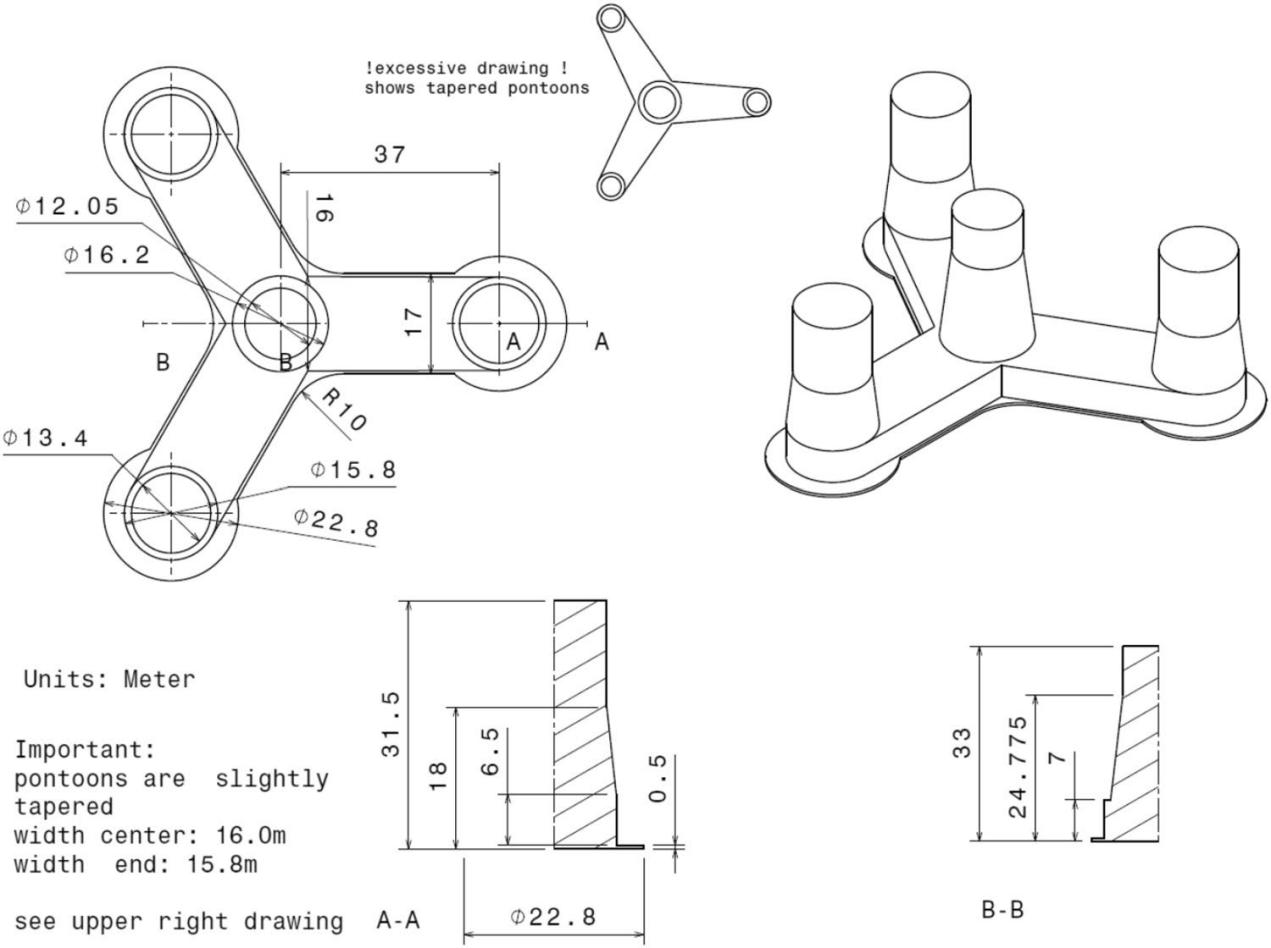
OO-Star floater 10-MW FWT’s main structural dimensions^13^.

**Figure 5:**
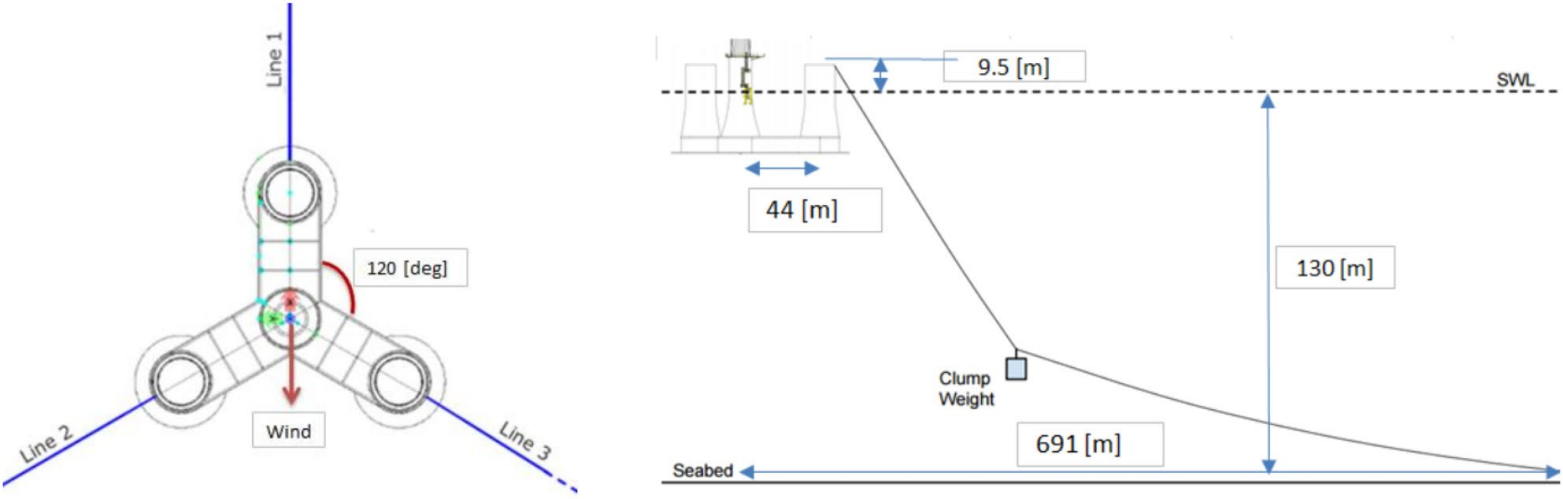
Sketch of 10-MW FWT’s mooring system, (left: top view; right: side view)^23^.

More OO-Star Wind Floater structural details being shown in Table 2 and Figs. 4 and 5.

**Table 2:** 10-MW OO-Star FWT’s wind floater main properties.

Parameters	Values
Water depth (m)	130.0
Draft (m)	22.0
Tower-base interface, above the mean sea level (m)	11.0
Displacement (tones)	24.2
Overall gravity, including ballast (tones)	21.7
Roll, pitch inertia about center of gravity (tones ∙m^2^)	1.4 × 10^7^
Yaw inertia about center of gravity (tones ∙m^2^)	1.6 × 10^7^
Center of gravity height below mean sea level (m)	15.2
Center of buoyancy height, below mean sea level (m)	14.2

In this study, a fully coupled 10-MW FWT aero-hydro-elastic-servo dynamic analysis has been conducted using the open-source FWT simulation program FAST (Fatigue, Aerodynamics, Structures Turbulence), created by the NREL (National Renewable Energy Laboratory). FAST code combines the following four computer codes: AeroDyn, HydroDyn, ServoDyn^24^. It is necessary to represent aerodynamic loads on the rotor blades, hydrodynamic loads acting on FWT floaters, control dynamics, FWT structural dynamics, and mooring system dynamics. Additionally, FAST provides a reading interface for time-varying stochastic winds in time-domain simulations. FAST has been successfully used in OC3: Offshore Code Comparison Collaboration and other well-known projects^25^. See also OC4: IEA Task Wind 30^26^, along with reported the validity of modeling has been confirmed using several floating constructions in the Netherlands, EU^27^. FWT tower bottom fore-aft bending moment (TwrBsMyt) was utilized as the case study in this work.

## Method

Probability distributions, as well as mean up-crossing rate function tails extrapolation have been successfully used for a range of offshore engineering applications in the past^17,28^. This study, however, enhances extrapolation by using deconvolution. Let $M\left( T \right) = {\text{max }}\{ \sigma \left( t \right):0 \le t \le T\}$ be the response process’s $\sigma \left( t \right)$ extreme value during a long-term time interval of length $T$, where $T$ is the planned FPSO operational service life, expressed in years. Different sea/environmental states certainly impact the stress response process $\sigma \left( t \right)$. Therefore, ergodicity should be assumed in order to draw statistical inferences from measurable data. For the up-crossings of high response levels under the assumption of a non-homogenous Poisson process, the long-term extreme value distribution of $M\left( T \right)$ is given as

1 $${\text{Prob}}\left( {M\left( T \right) \le \sigma } \right) \approx {\text{exp }}( - \nu^{ + } \left( \sigma \right)\,T)$$

where $\nu^{ + } \left( \sigma \right)$ denotes the long-term average up-crossing rate of the stress range $\sigma$. Well-known Rice formula for the mean up-crossing rate is as follows

2 $$\nu^{ + } \left( {\sigma |W} \right) = \mathop \smallint \limits_{0}^{ + \infty } zg_{{\sigma ,\dot{\sigma }}} \left( {\sigma ,\dot{\sigma }|W} \right)d\dot{\sigma }$$

with $W$ indicating a particular stationary sea state encountered by FPSO, and $g_{{X,\dot{X} }}$ being the joint PDF corresponding to the marginal PDF $f$^29–36^, with $\sigma$ being in this case FWT base VM stress, measured in MPa. Let’s examine now the distribution tail behaviour of the marginal complementary CDF (cumulative density function) $\overline{F}\left( \sigma \right)$. The same Poisson assumption of separate occurrences (namely, stress range exceedances) over a high threshold $\sigma \ge \sigma_{0}$, specifically in the tail, can be used to rewrite Eq. (1)

3 $${\text{Prob}}\left( {M\left( T \right) \le \sigma } \right) \approx {\text{exp }}\left( { - \overline{F}\left( \sigma \right)} \right),\quad \overline{F}\left( \sigma \right) \equiv \mathop \smallint \limits_{\sigma }^{\infty } f\left( s \right)ds$$

The analogous CDF function $\overline{F}\left( \sigma \right)$ can be extrapolated directly using novel deconvolution method. Stress range distribution in question belongs to a certain tail equivalence class

4 $$\nu^{ + } \left( \sigma \right)\, \propto \overline{F}\left( \sigma \right), \sigma \ge \sigma_{0}$$

The complementary CDF function $\overline{F}\left( \sigma \right)$ will also be used in this article to extrapolate distribution’s functional tail in the stress range $\sigma \ge \sigma_{0}$. In the following, deconvolution technique being described for the purpose of PDF tail extrapolation, note that the same technique is applicable to the complementary CDF tail. Consider stationary stochastic process $X\left( t \right)$, being either measured, or simulated over representative time lapse $0 \le t \le T$ and let one assume it may be represented as the sum of two separate stationary processes $X_{1} \left( t \right)$ and $X_{2} \left( t \right)$, namely

5 $$X\left( t \right) = X_{1} \left( t \right) + X_{2} \left( t \right)$$

It should be noted that our goal now is to present a general-purpose extrapolation technique that can be used to predict extreme values for a variety of dynamic systems. There are 2 alternative techniques to generate the marginal PDF $p_{X}$ for the process of interest $X\left( t \right)$:

A) directly extract $p_{X}^{A}$ from the underlying dataset, i.e., time series $X\left( t \right)$,
B) applying convolution $p_{X}^{B} = {\text{conv}}\left( { p_{{X_{1} }} , p_{{X_{2} }} } \right)$ after individually extracting PDFs from the process components $X_{1} \left( t \right)$ and $X_{2} \left( t \right)$, respectively $p_{{X_{1} }}$ and $p_{{X_{2} }}$.

The desired PDF $p_{X}$, being hence approximated by both $p_{X}^{A}$ and $p_{X}^{B}$. Although Method B) would give a more precise estimate of the desired PDF $p_{X}$, Method A) is easier to employ. The fact that convolution allows extrapolation of the empirical PDF $p_{X}^{A}$ that was directly extracted from case B) without assuming any specific extrapolation functional class, such as GEV (generalized extreme value distributions), required to extrapolate PDF tail towards design low probability levels of interest, is a benefit of using convolution scenario. It should be noted that the majority of extrapolation methods now in use, which are widely used in engineering practice, do actually rely on assuming specific extrapolation-functional classes. In practice, 2 independent component representations, provided by Eq. (5), being rarely available, hence one may search for artificial methods to estimate $p_{{X_{1} }}$ and $p_{{X_{2} }}$, or in the simplest situation, locate 2 identically distributed random process components $X_{1} \left( t \right)$ and $X_{2} \left( t \right)$ with $p_{{X_{1} }} = p_{{X_{2} }}$. The latter option, where processes $X_{1} \left( t \right)$ and $X_{2} \left( t \right)$ being equally dispersed, will be preferred in the following. Hence, the objective now would be to assess component-distribution $p_{{X_{1} }}$ such that given directly estimated PDF $p_{X}$ as in the option A)

6 $$p_{X} = {\text{conv}}\left( { p_{{X_{1} }} , p_{{X_{1} }} } \right)$$

hence limiting our following analysis to the deconvolution example alone. The region, where 2 vectors, ${\varvec{u}}$ and ${\varvec{v}}$ overlap, defines convolution of 2 vectors. Convolution being hence algebraically comparable to polynomial multiplication, whose coefficients being elements of ${\varvec{u}}$ and ${\varvec{v}}$. Suppose $m = {\text{length}}\left( {\varvec{u}} \right)$ and $n = {\text{length}}\left( {\varvec{v}} \right)$. Consequently, ${\varvec{w}}$ being the vector with length $m + n - 1$ and element $k$-th being

7 $$w\left( k \right) = \mathop \sum \limits_{j = 1}^{m} u\left( j \right)v\left( {k - j + 1} \right)$$

with summation going over all *j*-values that result in permissible subscripts for $u\left( j \right)$ and $v\left( {k - j + 1} \right)$, especially $j = {\text{max}}\left( {1,k + 1 - n} \right):1:{\text{min}}\left( {k,m} \right)$, when $m = n$, as will be the main case in this study, yielding

8 $$\begin{array}{*{20}c} {w\left( 1 \right) = u\left( 1 \right) \cdot v\left( 1 \right)} \\ {w\left( 2 \right) = u\left( 1 \right) \cdot v\left( 2 \right) + u\left( 2 \right) \cdot v\left( 1 \right)} \\ {w\left( 3 \right) = u\left( 1 \right) \cdot v\left( 3 \right) + u\left( 2 \right) \cdot v\left( 2 \right) + u\left( 3 \right) \cdot v\left( 1 \right)} \\ \cdots \\ {w\left( n \right) = u\left( 1 \right) \cdot v\left( n \right) + u\left( 2 \right) \cdot v\left( {n - 1} \right) + \cdots + u\left( n \right) \cdot v\left( 1 \right)} \\ \cdots \\ {w\left( {2n - 1} \right) = u\left( n \right) \cdot v\left( n \right)} \\ \end{array}$$

When the index grows from $n + 1$ to $2n - 1$, the reduced sections of the ${\varvec{w}}$-components are given by Eq. (14), ${\varvec{u}} = {\varvec{v}} = \left( {u\left( 1 \right), \ldots ,u\left( n \right)} \right)$. The latter doubles the initial distribution support domain and extends vector ${\varvec{w}}$ into the support domain. In brief, $\left( {2n - 1} \right) \cdot \Delta x \approx 2n \cdot \Delta x = 2X_{L}$ means that the distribution support length has been doubled. The constant length of each discrete distribution bin in this example is $\Delta x$, and when compared with the original distribution support length, $n \cdot \Delta x = X_{L}$. $\Delta x$. Convolution convects distribution features further along the PDF tail. ${\varvec{w}} = \left( {w\left( 1 \right), \ldots ,w\left( n \right)} \right)$, where n is the length of the distribution support $\left[ {0,X_{L} } \right]$, represents the empirical target distribution $p_{X}$. To reduce complexity, only one-sided positive random variables, $X \ge 0$, being taken into consideration in this study. According to Eq. (8), the distributions for the vectors ${\varvec{w}}$ and ${\varvec{u}}$ are $p_{X}$ and $p_{{X_{1} }}$, respectively. The supplied ${\varvec{w}} = \left( {w\left( 1 \right), \ldots ,w\left( n \right)} \right)$ in Eq. (13) may be used to determine the unknown components ${\varvec{u}} = {\varvec{v}} = \left( {u\left( 1 \right), \ldots ,u\left( n \right)} \right)$. It begins with the 1st component $u\left( 1 \right) = \sqrt {w\left( 1 \right)}$, moves on to the 2nd component $u\left( 2 \right) = \frac{w\left( 2 \right)}{{2u\left( 1 \right)}}$, and continues, until n reaches last component $u\left( n \right)$. This technique allows for straightforward extrapolation of self-deconvoluted vector $\left( {u\left( 1 \right), \ldots ,u\left( n \right)} \right)$ towards $\left( {u\left( {n + 1} \right), \ldots ,u\left( {2n - 1} \right)} \right)$. In essence, the tail of $p_{{X_{1} }}$ extrapolates linearly throughout the range $\left( {X_{L} ,2X_{L} } \right)$. The $p_{{X_{1} }}$ is now referred to as a deconvoluted distribution, described by projected vector ${\varvec{u}}$ in its discrete form. The vector ${\varvec{w}}$ being lengthened and projected to double the initial distribution support domain based on Eq. (8). In summary, compared to the initial PDF support length $n \cdot \Delta x = X_{L}$, the $p_{X}$ PDF support length is twice, $\left( {2n - 1} \right) \cdot \Delta x \approx 2n \cdot \Delta x = 2X_{L}$. Deconvolution extrapolation does not require any particular extrapolation functional class, as has been described in the Introduction, in order to extrapolate the distribution functional tail. Since it is more crucial to estimate the probability of exceedance, or $\overline{F} =$ 1 $-$ CDF (where CDF stands for cumulative density function), rather than the marginal PDF, in the majority of reliability analysis engineering applications, the probability of exceedance $\overline{F}$ will be further denoted by the notation $f_{X}$ in this section, whose PDF tail will be extrapolated in the same way as the marginal probability density function PDF $p_{X}$ from Eq. (6). The suggested technique, however, could be appropriate for any sufficiently regular, monotonically declining concave or convex functional PDF tail.

The "shorter" version of the original data set has been extrapolated for the purpose of comparison with forecasts based on the entire "longer" data set in order to validate the extrapolation approach indicated above. Hence, the purpose of this study was to demonstrate that the recommended extrapolation approach is at least a few orders of magnitude more efficient. The goal now is to assess deconvoluted PDF $f_{{X_{1} }}$, obtained from the empirical PDF $f_{X}$, being based on sequentially solving Eq. (8). It appears that the final values of the resultant vector ${\varvec{u}}$, say $\left( {u\left( {n - L} \right), \ldots ,u\left( n \right)} \right)$ for some $L < n$, may become negative, since the deconvoluted values ${\varvec{u}} = \left( {u\left( 1 \right), \ldots ,u\left( n \right)} \right)$ often follow a monotonously declining pattern (same was anticipated for the empirical parent PDF $f_{X}$). Due to the fact that PDFs may only be represented by positive numbers, the latter is obviously a numerical mistake, and cannot be accepted. The following scaling approach has been devised by authors to address that numerical problem. The pivot value is chosen to be the lowest positive value $f_{L}$ of the provided PDF tail of $f_{X}$. Hence, scaling is only a linear adjustment along the distribution's vertical *y*-axis on the decimal-logarithmic scale

9 $$g_{X} = { }\mu \left( {{\text{log}}_{{{10}}} \left( { f_{X} } \right) - {\text{log}}_{{{10}}} \left( { f_{L} } \right)} \right) + {\text{log}}_{{{10}}} \left( { f_{L} } \right)$$

having reference level $f_{L}$ being unaltered and $g_{X} \left( x \right)$ being a scaled ${\text{log}}_{{{10}}}$ version of the empirical base PDF $f_{X}$. To conveniently prevent the formation of negative components in the resultant $f_{{X_{1} }}$, the scaling coefficient $\mu$ is set to be $1/3$ worked nicely for both numerical situations, examined in this study. Then, after finding $f_{{X_{1} }}$ and performing back convolution $\tilde{f}_{X} = {\text{conv}}\left( { f_{{X_{1} }} , f_{{X_{1} }} } \right)$, $f_{{X_{1} }}$, as in Eq. (6) can be completed, the original scale will be restored by performing inverse scaling using $\mu^{ - 1}$ with $\tilde{f}_{X}$ being an extrapolated version of $f_{X}$. As discussed in^13–17,37,38^, numerous offshore engineering solutions have been extrapolated using novel deconvolution method^39,40^; this method provides the following 4-parametric form for the tail mean up-crossing rate function

10 $$\nu^{ + } \left( \sigma \right) \approx q \cdot {\text{exp}}\left( { - a\left( {\sigma - b} \right)^{c} } \right) ,\quad \sigma \ge \sigma_{0}$$

with $\sigma$ being the response level, which is the stress in the case of this paper; $a, b, c, q$ being 4 suitable PDF tail constants; $\sigma_{0}$ is a suitable tail marker, indicating the start of the fit based on Eq. (10). In the case of $q = c = 1$, this simply represents the GEV distribution, Gumbel type. Mean up-crossing rate function $\nu^{ + }$ is often used in combination with the Poisson assumption, namely that extreme events (PDF tail range), crossing high thresholds can be approximated as independent.

Thus, it is anticipated that near-to-complete linear tail behaviour will be achieved by graphing ${\text{ln}}\left\{ {{\text{ln}}\left( {\nu^{ + } \left( \sigma \right)/q} \right)} \right\}$ versus ${\text{ln}}\left( {\sigma - b} \right)$. The mean square error function *F* with respect to the 4 inputs $a, b, c,d$ is to be minimized in order to do the optimization on the decimal log level

11 $$F\left( {a, b, c,d} \right){ = }\mathop \smallint \limits_{{\sigma_{0} }}^{{\sigma_{1} }} w\left( \sigma \right)\left\{ {{\text{ln}}\left( {\nu^{ + } \left( \sigma \right)} \right) - \ln q + a\left( {\sigma - b} \right)^{c} } \right\}^{2} d\sigma$$

where $\sigma_{1}$ being a suitable data cut-off value, namely, the largest response value, from which the width of the confidence interval can be determined. The definition of the weight function w is given as follows: $w\left( \sigma \right){ = }\left\{ {{\text{ln}}C^{ + } \left( \sigma \right) - {\text{ln}}C^{ - } \left( \sigma \right)} \right\}^{ - 2}$ where $\left( {C^{ - } \left( \sigma \right), C^{ + } \left( \sigma \right)} \right)$ with a 95% confidence interval (CI), which was empirically derived from measured data. In offshore, naval and marine engineering, novel deconvolution extrapolation has been proved to be a reliable and effective extrapolation technique for a broad variety of random processes. The series of conditional exceedances above a threshold $\lambda$ for any generally ergodic wave height or wind speeds and wave heights process may be considered to be a Poisson process, but generally one that isn't homogenous. As a result, the approximate boundaries of a *p-*% CI (confidence interval) of $p_{k} \left( \lambda \right)$ may be determined for levels $\lambda$ of approaching 1

12 $${\text{CI}}^{ \pm } \left( \lambda \right) = p_{k} \left( \lambda \right)\left( {1 \pm \frac{f\left( p \right)}{{\sqrt {\left( {N - k + 1} \right)p_{k} \left( \lambda \right)} }}} \right)$$

$f\left( p \right)$ was calculated using the inverse normal distribution, with values such as $f\left( {90\% } \right) = 1.65$, $f\left( {95\% } \right) = 1.96$. $N$ is the overall number of local maxima that were built in the examined vector $\vec{R}$. Engineering reliability tasks that require accurate extreme value prediction are frequent and difficult, especially when there is a lack of data. Hence, it is important from a practical design standpoint to create new, effective, and precise extrapolation approaches. For practical engineering example of the above-discussed issues, see Sect. 4.3, where the VM stress range PDF tail along with fatigue damage assessment are presented.

## Numerical results

This section discusses the numerical results of the fatigue damage calculation for the taken into account FWT tower bottom fore-aft bending moment. We briefly cover the analyzed load scenarios as well as the suitable material properties for determining FWT fatigue damage.

### Load cases along with environmental conditions

In this study, the winds and waves statistics were built using hindcast data that was obtained from the North Sea between years 2001 and 2010. The long-term combined wind and wave distribution was made up of the 1-h mean wind speed, which was located 10 m above sea level (*U*_10_), the wave spectral peak period (*T*_*p*_) along with the significant wave height (*H*_*s*_)^41^. The long-term joint wind-wave PDF was

13 $$f_{{U_{10,} H_{s,} T_{p} }} \left( {u,h,t} \right) = f_{{U_{10} }} \left( u \right) \cdot f_{{H_{s} \left| {U_{10} } \right.}} \left( {h\left| u \right.} \right) \cdot f_{{T_{p} \left| {U_{10, } H_{s} } \right.}} \left( {t\left| {u,h} \right.} \right)$$

where the marginal distribution of *U*_10_ may be described by $f_{{U_{10} }} \left( u \right), f_{{H_{s} \left| {U_{10} } \right.}} \left( {h\left| u \right.} \right)$ and $f_{{T_{p} \left| {U_{10, } H_{s} } \right.}} \left( {t\left| {u,h} \right.} \right)$, the conditional PDF of *H*_*s*_ for a given *U*_10_ and conditional distribution of *T*_*p*_ for a given *U*_10_ and *H*_*s*_.

For the purpose of simulating the FWT typical operating in-situ conditions, 3 representative load cases have been selected, see Table 3. Using in-situ the joint PDF, wave heights and spectra peak periods for each wind speed have been calculated following Eq. (13). The wind speed profile was modelled using conventional wind power-law formulation

14 $$U_{w} \left( z \right) = { }U_{hub} { }\left( {\frac{{\text{Z}}}{{Z_{hub} }}} \right)^{\alpha }$$

with *U*_*w*_*(z)* being mean wind speed, measured at elevation $z$ above the still water level, *u*_*hub*_ being mean wind speed w.r.t hub elevation, *z*_*hub*_ being hub elevation w.r.t the still water level (119 m for selected 10-MW FWT). *α (*power-law exponent) being equal to 0.14, according to recommendations IEC 61,400–3-2^42^. The 3-D wind turbulent fields were generated using TurbSim, being derived from Kaimal's turbulence model^43^. The JONSWAP (Joint North Sea Wave Project) spectrum allowed proper modelling of time-varying irregular waves, having specific *H*_*s*_ and *T*_*p*_ values. 4000 s have been spent on each simulation; the first 400 s of these simulations were disregarded in order to account for the transient impact that is commonly present during a turbine's beginning. To assess the extreme value, only remaining 3600 s (1 h) were used. Therefore, each environmental factor contained 20 random wave and wind condition samples in addition to the various sea conditions.

**Table 3:** Numerical MC simulation load cases.

Load cases	$U_{w}$ (m/s)	$T_{I}$	$H_{s}$ (m)	$T_{p}$ (s)	MC samples	MC sample simulation length (hours)
LC1	8	0.1740	1.9	9.7	20	1
LC2	12	0.1460	2.5	10.1	20	1
LC3	16	0.1320	3.2	10.7	20	1

### S–N curve

According to DNV GL class recommendations, the fatigue design S–N curves from the fatigue testing reported in this section were selected^44–47^. The mean-minus-two-standard-deviation curves serve as the foundation for the S–N curves employed in this design, which have a 97.6% survival probability^44–48^

15 $${\text{log}}N = {\text{log}}\overline{a} - m \cdot {\text{log}}\left( {\Delta \sigma \left( {\frac{t}{{t_{ref} }}} \right)^{k} } \right)$$

with $t = 75$ mm, $t_{ref} = 25$ mm, $k = 0.2$ and S–N curve parameters given in Table 4.

**Table 4:** D (FAT 90) material S–N parameters.

S–N curve	Material	$N \le 10^{7}$		$N \ge 10^{7}$	
		${\text{log}}\overline{a}$	$m$	${\text{log}}\overline{a}$	$m$
D (FAT 90)	Welded joint	12.164	3.0	15.606	5.0

The hot spot D curve has been used in combination with the stress measurements, to model welded details. An additional stress concentration and thickness effects may be relevant, to more accurately represent realistic butt welds, and deck outfitting details.

**Figure 6:**
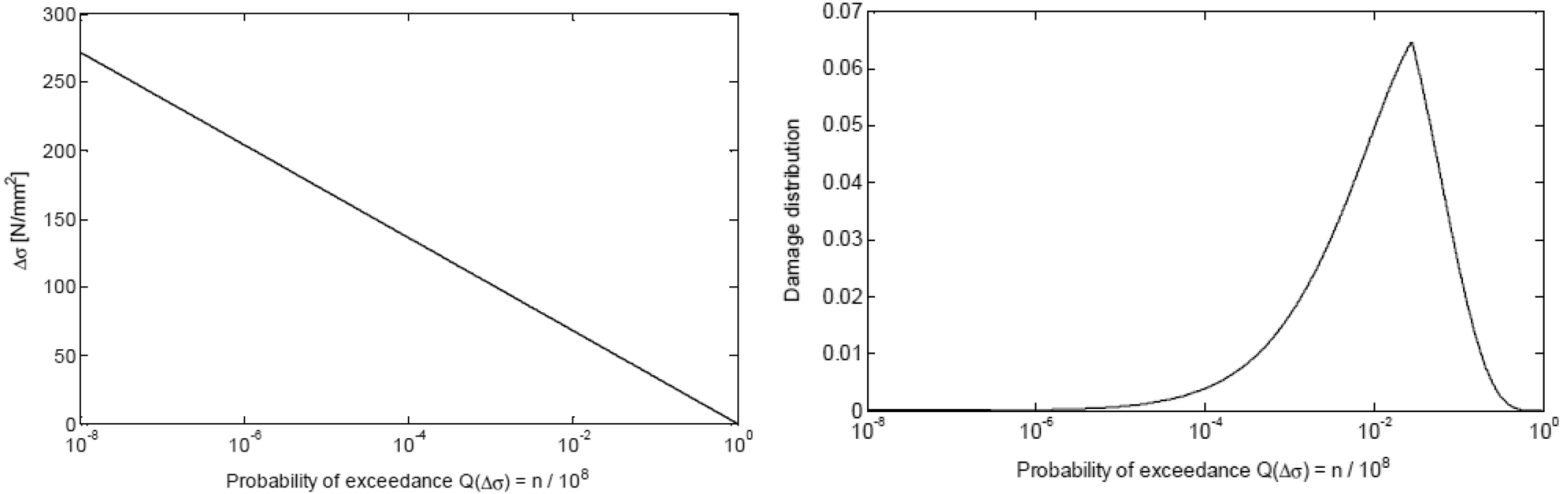
Left: Straight line marks spectrum yields damage of 1.0 was based on S–N curve D (FAT 90). Right: Damage distribution from straight line spectrum was based on S–N curve D (FAT 90)^47^.

Figure 6 presents a straight line spectrum, with yielding fatigue damage of 1.0, based on S–N curve D/FAT 90 on the left; damage PDF for a linear line spectrum was based on S–N curve D/FAT 90 on Fig. 6 right^46,47^. When the power parameter $m$ in Eq. (15) being large, fatigue contribution will come from a PDF tail at exceedance probability levels, below 10^−2^, meaning that accurate PDF tail extrapolation being essential for accurate fatigue assessment^49–59^.

### Fatigue damage distribution tail importance

According to the Palmgren–Miner's rule, the S–N fatigue approach was used to calculate the fatigue damage brought on by changing loads under the premise of linear cumulative damage^46^. The total fatigue damage, experienced by FWT structure has been modeled as the accumulation of each load cycle's damage at various VM stress levels, regardless of the sequence in which the VM stress cycles occur. When a VM stress histogram comprised of many useful blocks of constant amplitude is used to illustrate the long-term VM stress range distribution, $\Delta \sigma_{i}$ each with VM stress consequent repetitions $n_{i}$

16 $$D = \mathop \sum \limits_{i = 1}^{k} \frac{{n_{i} }}{{N_{i} }} = \frac{1}{{\overline{a}}}\mathop \sum \limits_{i = 1}^{k} n_{i} \left( {\Delta \sigma_{i} } \right)^{m} \propto - \mathop \smallint \limits_{0}^{ + \infty } \sigma^{m} d\overline{F}\left( \sigma \right) = m\mathop \smallint \limits_{{\sigma_{0} }}^{ + \infty } \sigma^{m - 1} \overline{F}\left( \sigma \right)d\sigma = \mathop \smallint \limits_{0}^{ + \infty } \sigma^{m} f\left( \sigma \right)d\sigma$$

with $D$ accumulated fatigue damage, $\overline{a}, m$ S–N fatigue parameters, *k* number of VM stress blocks, $n_{i}$ number of VM stress cycles within block/bin $i$, $N_{i}$ number of cycles to failure given constant VM stress range $\Delta \sigma_{i}$.

$\overline{F}\left( \sigma \right) \equiv 1 - F\left( \sigma \right)$ complemantary rainflow VM stress range cumulative density function (CDF). See Section "Method" for details regarding the integrand $\overline{F}\left( \sigma \right)$ in Eq. (3), $f\left( \sigma \right) = F^{\prime } \left( \sigma \right)$. In Eq. (16) linear fatigue accumulation assumption was used, and that on itself being a simplification. Note that in this study the underlying response distribution $\overline{F}$ was the major focus, rather than fatigue accumulation method itself. To assess how the PDF tail impacts the overall fatigue damage^60–72^, one can assess separately fatigue damage tail damage part $D_{{{\text{tail}}}}$

17 $$D_{{{\text{tail}}}} = m\mathop \smallint \limits_{{\sigma_{0} }}^{ + \infty } \sigma^{m - 1} \overline{F}\left( \sigma \right)d\sigma$$

based on the assumption that only ranges of cyclic VM stresses need to be taken into account^44–47^. In this section, fatigue damage for VM stress range distribution was estimated by the rainflow counting. In order to estimate the relevance of the distribution tail for measuring fatigue damage, one has to at least approximately define the distribution tail region, specifically the tail cut-on VM stress value, $\sigma_{0}$ and cut-off value $\sigma_{1}$. To evaluate how significant the PDF tail is, for assessing fatigue damage, one may:

A. approximately identify probability distribution tail region, i.e., tail cut-on stress value $\sigma_{0}$;
B. observe fatigue damage variation with reduction of the distribution tail, i.e., introducing tail cut-off value $\sigma_{1}$;
C. validate method by using reduced data record (for example by taking only 100th data point) with a shorter distribution tail that can be confidently cut off at the response threshold $\sigma_{1}$, with subsequent use of tail extrapolation and fatigue damage correction

with PDF (or complementary CDF = $\overline{F}$) functional tail cut-on, and cut-off stress values $\sigma_{0}$ and $\sigma_{1}$ being dependent on a particular underlying dataset. An equidistant selection from the whole dataset was utilized to produce the smaller dataset with the same statistics. Remember that the total simulated time $T$ Both full dataset and the reduced dataset (obtained from the full dataset by taking only each 100th data point), resulting in a 60-h numerical simulation, accounting for in-situ wind speeds scatter diagram. Hence, advocated method has been validated, by using complete and reduced datasets, and then comparing corresponding full and extrapolated fatigue damage.

**Figure 7:**
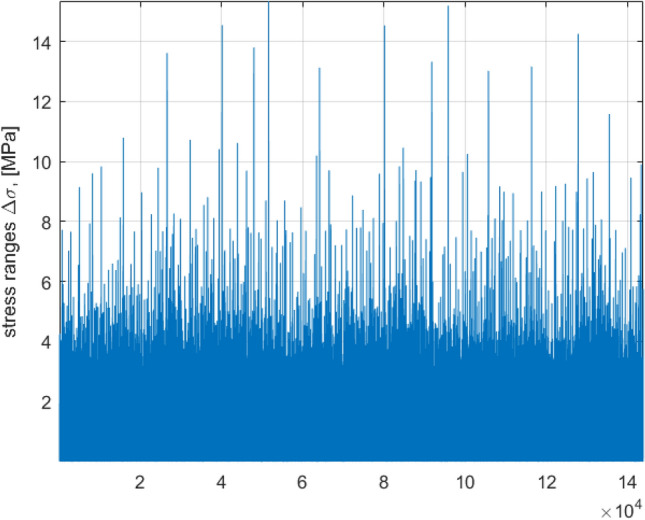
Stress ranges, obtained by rainflow counting.

Figure 7 presents raw MC simulated data for stress ranges $\sigma$, obtained by rainflow counting.

**Figure 8:**
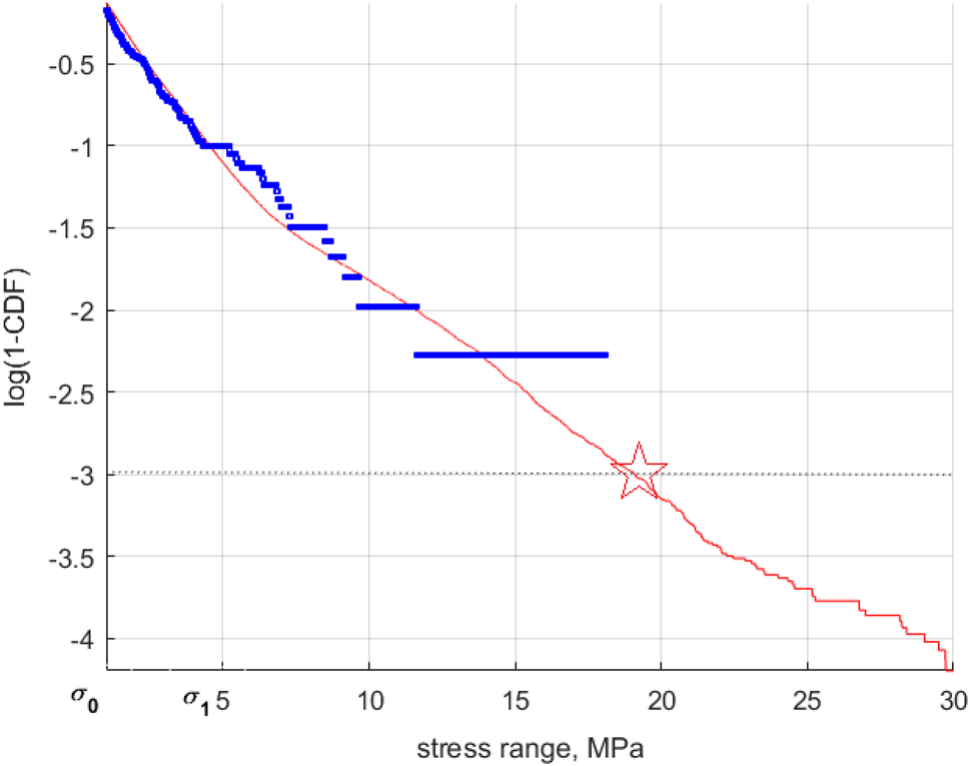
Decimal logarithmic CDF tail of the rainflow VM stress range’s complementary CDF: full dataset (–); reduced dataset (*). Arrows indicate cut-on, cut-off tail stress markers $\sigma_{0}$, $\sigma_{1}$(about 1, and 4 MPa respectively). Star marks VM stress range full dataset at 10^−3^ exceedance probability level.

Figure 8 presents decimal logarithmic tail of the rainflow VM stress range exceedance $\overline{F} =$ 1-CDF for FWT tower-base VM stress. VM stress range values $\sigma_{0}$ and $\sigma_{1}$ may be interpreted as PDF/CDF tail markers. After assessing.

A. full damage $D$ per simulation, according to Eq. (10)
B. reduced damage $D_{{{\text{reduced}}}}$ based on same distribution $\overline{F}\left( \sigma \right)$ as the total damage $D$, but with a cut tail, i.e., $\overline{F}\left( \sigma \right) = 0, \sigma > \sigma_{1}$.

the resulting conclusion is $D_{{{\text{reduced}}}} > 5D$. Hence, while projecting cumulative fatigue damage, it is necessary to properly estimate the tail of the distribution. In other words, reduced underlying dataset substantially overestimated fatigue damage, suggesting that proper tail extrapolation is essential for reliable fatigue estimations.

### Fatigue damage assessment using exceedance probability tail extrapolation

This section calibrates the recommended extrapolation method using partially observed data. Predictions are then made using the calibrated extrapolation model. The remaining observable data are then used to assess how well the forecasts performed. As was mentioned before Section "Method", PDF tail may include significant fatigue damage weight $D_{{{\text{tail}}}} /D$ from Eqs. (16), (17). Thus, accurate PDF tail extrapolation according to Eq. (10), enabling accurate estimation of both fatigue damage, as well as FWT fatigue life.

**Figure 9:**
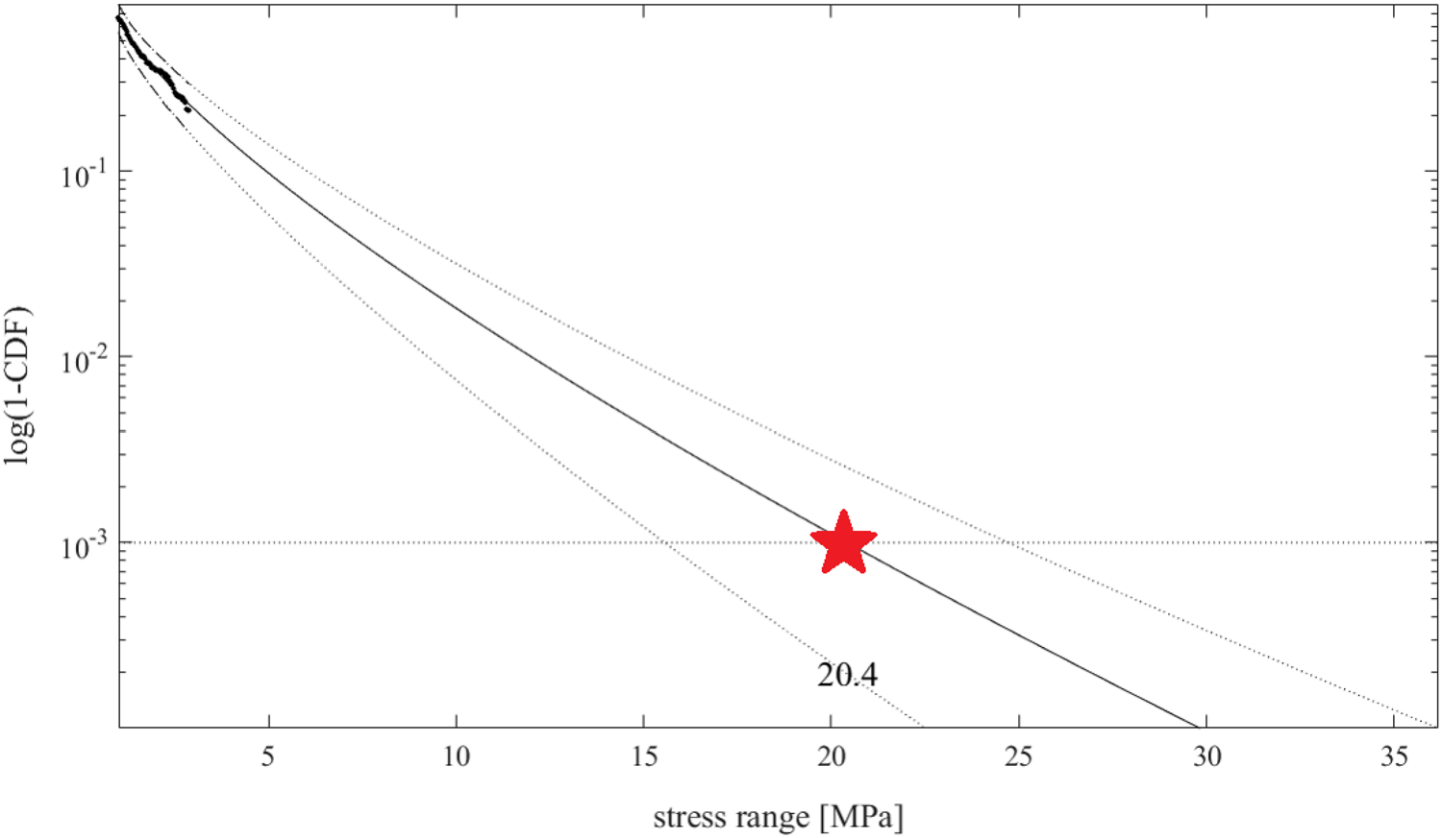
Decimal logarithmic tail of rainflow VM stress range 1-CDF reduced dataset (*); extrapolated 1-CDF tail (–). Star marks predicted value.

Figure 9 presents decimal logarithmic VM stress range PDF tail: namely ($\cdot$) full dataset; (*) reduced dataset; (–) $\overline{F} \equiv$ 1-CDF tail extrapolated according to Eq. (10), optimized as in Eq. (11), and described in Section "Method". Longitudinal VM stress range being plotted in MPa on the horizontal axis, PDF decimal logarithm values being presented on the vertical axis. Figure 9, compared to Fig. 6 shows that the set VM stress range level at 10^−3^ according to comparison of the whole dataset and the reduced extrapolated one, the exceedance probability level is correctly assessed. The latter indicates that fatigue damage, which was first overestimated by reduced dataset analysis, has been "restored" by proper extrapolation approach. $D_{{{\text{tail}}}} /D$, from Eqs. (16), (17), see Section "Method" has been reduced from 5 down to 1.4, showing that advocated methodology is effective. The latter aspect being crucial for engineering structural design, seen from practical standpoint.

Rainflow counting was done on VM stresses (positive), hence differences in tensile and compressive stress cycles, as well as mean stress correction have been ignored. Note, however, that this study was focused on introducing fatigue distribution extrapolation technique, and not on the fatigue rainflow counting model itself, since any preferred fatigue rainflow counting model can be coupled to the advocated fatigue distribution extrapolation method. This study presented generic fatigue damage/life assessment method, hence both rainflow counting model and fatigue spot location were chosen purely exemplary, to provide advocated method illustration.

## Conclusions

Due to the importance of FWTs in modern renewable energy industry, safety and reliability are concerns that are regularly faced in practice and need for accurate and reliable design and monitoring procedures. In this study, the methodology for estimating the fatigue damage to the 10 MW DTU WT-OO-Star under real operating conditions is described. The approach described here may be used to generate FWT settings that would minimize potential fatigue damages during the primary FWT design phase. If the latter metrics are available, it is imperative to compare analytical and numerical engineering techniques to observable data.

The FWT tower base VM stress being the underlying dataset, employed in this study to assess accuracy of advocated fatigue damage calculation method. Apart from stress range distribution inaccuracy itself, fatigue estimations' uncertainty has other important uncertainties, for example using specific fatigue accumulation damage method bears its own simplifications. Typically, stress range distribution tail plays secondary role. This study shows, however, that the stress range distribution tail may have significant influence on the overall fatigue damage. Therefore, in FWT reliability analysis, lowering the uncertainty, linked to the extreme tail of the fatigue estimate is of practical importance. In other words, having underlying dataset, being too short, may result in non-conservative estimates of fatigue life and underestimating of fatigue damage.

This study has shown that probability distribution functional tail may still contain considerable fatigue damage, which might contribute for more than half of the total fatigue damage. The latter is obviously important for engineering since it can prevent a significant underestimation of fatigue damage by addressing a data scarcity with relation to fatigue evaluation. Engineers must carefully consider these aspects since it is manifestly unconservative to underestimate fatigue damage or overestimate fatigue life.

The recommended extrapolation technique correctly recovered fatigue damage from data that was 500-times shorter than the total dataset. The latter emphasizes the precision and applicability of the offered method. It should be highlighted that because some crucial factors have been ignored, the linear accumulation assumption alone is a highly illogical approach to estimate fatigue damage. It is crucial to remember that, despite the linear accumulation assumption being the basis for the recommended fatigue calculation results, the study's main focus has been on the underlying response/load distribution rather than how to measure the subsequent fatigue damage. In other words, the more accurate underlying response/load probability distributions, such the rainflow VM stress range probability distribution in the analyzed case, would nevertheless be beneficial, given any alternative, more precise non-linear fatigue accumulation assumption.

Finally, it is important to mention that generic fatigue assessment method, presented here, by no means limited to a particular offshore wind turbine structure, studied here.

## Data Availability

The raw/analyzed data from this study is available on request from Dr. Oleg Gaidai, o_gaidai@just.edu.cn.
